# De Novo Transcriptome Profiling for the Generation and Validation of Microsatellite Markers, Transcription Factors, and Database Development for *Andrographis paniculata*

**DOI:** 10.3390/ijms24119212

**Published:** 2023-05-24

**Authors:** Rakesh Singh, Akshay Singh, Ajay Kumar Mahato, Ritu Paliwal, Gunjan Tiwari, Ashok Kumar

**Affiliations:** 1 Division of Genomic Resources, ICAR-National Bureau of Plant Genetic Resources, New Delhi 110012, India; 2 The Centre for DNA Fingerprinting and Diagnostics, Hyderabad 500039, India; 3 CSIR-Central Institute of Medicinal and Aromatic Plants, Lucknow 226015, India; 4 Division of Germplasm Evaluation, ICAR-National Bureau of Plant Genetic Resources, New Delhi 110012, India

**Keywords:** *Andrographis paniculata*, transcriptome, EST-SSRs, transcription factors

## Abstract

*Andrographis paniculata* belongs to the family Acanthaceae and is known for its medicinal properties owing to the presence of unique constituents belonging to the lactones, diterpenoids, diterpene glycosides, flavonoids, and flavonoid glycosides groups of chemicals. Andrographolide, a major therapeutic constituent of *A. paniculata,* is extracted primarily from the leaves of this plant and exhibits antimicrobial and anti-inflammatory activities. Using 454 GS-FLX pyrosequencing, we have generated a whole transcriptome profile of entire leaves of *A. paniculata*. A total of 22,402 high-quality transcripts were generated, with an average transcript length and N50 of 884 bp and 1007 bp, respectively. Functional annotation revealed that 19,264 (86%) of the total transcripts showed significant similarity with the NCBI-Nr database and were successfully annotated. Out of the 19,264 BLAST hits, 17,623 transcripts were assigned GO terms and distributed into three major functional categories: molecular function (44.62%), biological processes (29.19%), and cellular component (26.18%) based on BLAST2GO. Transcription factor analysis showed 6669 transcripts, belonging to 57 different transcription factor families. Fifteen TF genes that belong to the NAC, MYB, and bHLH TF categories were validated by RT PCR amplification. In silico analysis of gene families involved in the synthesis of biochemical compounds having medicinal values, such as cytochrome p450, protein kinases, heat shock proteins, and transporters, was completed and a total of 102 different transcripts encoding enzymes involved in the biosynthesis of terpenoids were predicted. Out of these, 33 transcripts belonged to terpenoid backbone biosynthesis. This study also identified 4254 EST-SSRs from 3661 transcripts, representing 16.34% of the total transcripts. Fifty-three novel EST-SSR markers generated from our EST dataset were used to assess the genetic diversity among eighteen *A. paniculata* accessions. The genetic diversity analysis revealed two distinct sub-clusters and all accessions based on the genetic similarity index were distinct from each other. A database based on EST transcripts, EST-SSR markers, and transcription factors has been developed using data generated from the present study combined with available transcriptomic resources from a public database using Meta transcriptome analysis to make genomic resources available in one place to the researchers working on this medicinal plant.

## 1. Introduction

*A. paniculata*, an annual dicotyledonous essential medicinal herb, belongs to the family Acanthaceae and is commonly known as “Kalmegh” in India. It is native to Asia and widely distributed in India, China, Sri Lanka, Cambodia, Malaysia, Thailand, and Taiwan [1]. The aerial parts of *A. paniculata* contain many bioactive compounds, such as lactones, diterpenoids, flavonoids, diterpenoids lactones, diterpenoid glycosides, and flavonoid glycosides [2,3,4,5]. Andrographolide is the major compound present in *A. paniculata* in terms of bioactive properties and abundance. Leaves contain the highest amount of andrographolide, while seeds contain the least [6]. *A. paniculata* is recommended as an anti-diabetic, immune stimulant, anti-microbial, anti-fungal, anti-viral, anti-inflammatory, anti-carcinogenic, anti-bacterial, anti-HIV, and anti-thrombotic [7,8,9]. Traditionally, *A. paniculata* has been used mainly for treating fever, diarrhea, flu, diabetes, liver disease, skin diseases, snake bite, and poisonous stings of insects [10]. Due to the presence of these important pharmacological properties, this plant is a top choice for modern pharmacopeia and conventional medical practitioners as a key ingredient for various polyherbal formulations in the traditional Indian systems of medicine (Ayurveda).

Recent advances in next-generation sequencing (NGS) are an important tool for generating high-throughput molecular data in a cost-effective manner, which provides opportunities to understand the complete outlook of the genomic and transcriptomic profile of any species [11,12]. A high-quality chromosome-scale assembly of the *A. paniculata* genome [13,14] and transcriptome sequences [8,15,16,17,18] is now available for the scientific community working on the *A. paniculata* genetic improvement. The availability of a reference genome and transcriptome sequences facilitates the identification of agronomically important genes that control yield as well as tolerance to biotic and abiotic stresses. The availability of RNA-Seq data can accelerate the development of EST-SSR markers, which can be useful for genetic diversity and population structure analysis, genetic linkage map construction, association mapping, marker-assisted selection (MAS) breeding, and quantitative trait locus mapping in non-model plant species [19,20,21].

Earlier, the genetic relationships and variability analysis among *A. paniculata* germplasms have been investigated using random amplified polymorphic DNA (RAPD) [22,23,24,25,26], isozyme [27], single-strand conformation polymorphism (SSCP) [28], amplified fragment length polymorphism (AFLP) and simple sequence repeats (SSR) markers [29], sequence-related amplified polymorphism (SRAP) and single nucleotide polymorphism (SNP) [30], start codon targeted (SCoT) and CAAT-box derived polymorphism (CBDP) markers [31], inter simple sequence repeats (ISSR) marker [32,33,34], and genomic SSR (g-SSR) [35]. For judicious exploitation of *A. paniculata* germplasm, comprehensive and robust DNA-based markers are required for an in-depth understanding of natural diversity and for developing strategies for its sustainable utilization. Therefore, the generation of a large-scale EST dataset is a useful approach to accelerating the research efforts of non-model plant species in the area of genomics and biodiversity studies [19].

Transcription factors (TFs) are sequence-specific DNA binding trans-regulatory proteins that play an important role in plant growth and development and responses against various biotic and abiotic stresses. They regulate gene expression by binding to specific cis-regulatory sequences in the promoters of their target genes [36,37]. Molecular markers and transcription factors from the transcriptome-based analysis have been generated for several plant species such as *Operculina turpethum* [38], *Chlorophytum borivilianum* [39], *Ocimum gratissimum* [40], *Abelmoschus esculentus* [41], *Platanus acerifolia* [42], and *Picea neoveitchii* Mast. [43], and transcription factor gene-derived microsatellite (TFGM) markers have also been developed in *Medicago truncatula* [44], Lilium species [45], and *Camellia sinensis* [46]. In recent years, there has been rapid progress in the generation of genomic resources based on transcriptome sequencing for several plant species, such as Banana (*Musa acuminata*) [47], Urdbean (*Vigna mungo*) [48], Noug (*Guizotia abyssinica*) [49], *Tinospora cordifolia* [50], and Chickpea (*Cicer arietinum*) [51].

In this paper, we report the generation of a large-scale EST dataset by capturing entire transcripts from young leaves of *A. paniculata* through 454 GS-FLX pyrosequencing techniques. The EST dataset was further exploited for the development of EST-SSR markers and the identification of transcription factors (TFs). We also developed an online database (ApTransDB) of EST-SSR markers and transcription factors for *A. paniculata*.

## 2. Results

### 2.1. 454 GS-FLX Sequencing and De Novo Transcriptome Assembly

Transcriptome sequencing from the leaves of *A. paniculata* generated a total of 748,409 single-end raw reads. After the quality check, trimming, and removal of adapter sequences, a total of 730,670 (97.62%) high-quality-filtered reads with an average read length of 500 bp were obtained (Figure S1). The filtered reads had a total length of 301,674,417 bp, and the GC content was 48% (Table 1). The Newbler assembly resulted in the generation of a total of 16,149 transcripts and 65,004 singletons. The transcripts generated by Newbler were 968 bp an average length, and N50 of 1184 bp. Therefore, to check the assembly status, high-quality trimmed reads (730,670) were mapped back to Newbler assembled transcripts using CLC Genomics Workbench; only 85.61% of the total quality reads were mapped. Further, mapping of the 65,004 singletons has been performed over the 16,149 Newbler-generated transcripts using CLC Genomics Workbench, which led to the discovery of 9328 duplicates. After removing duplicates, the remaining singletons were assembled using MIRA assembler v4.2. From such analyses, 6253 transcripts with an average length of 669 bp were generated, which were not assembled by the Newbler assembler. Finally, transcripts generated by Newbler v2.9 and MIRA v4.2 assembly software were merged into a single FASTA file that contained 22,402 transcripts with an average transcript length of 884 bp (Table 1).

Further, when high-quality filtered reads were mapped back to the final assembled transcripts, 89.33% of reads were mapped successfully. An addition of 3.2% more reads in the final assembly indicates the effectiveness of using both Newbler v2.9 and MIRA v4.2 assemblers in the assembly of the *A. paniculata* transcripts.

### 2.2. Functional Annotation and Classification of Transcripts

The final assembled transcripts were subjected to functional annotation to determine their possible functions. All 22,402 transcripts were compared against the non-redundant (nr) protein database (NCBI) using the BLASTX program with an E-value cut-off of 10^−5^. A total of 19,264 (86%) transcripts showed significant similarity with the NCBI non-redundant (nr) protein database and were successfully annotated, and the remaining 3138 (14%) transcripts showed no BLAST hits. The BLASTX similarity distribution of annotated transcripts (19,264) showed that the maximum number of transcript sequences had similarities in the range of 75% to 95% (Figure S2). The species distribution graph of annotated transcripts of the *A. paniculata* showed the highest similarity with *Mimulus guttatus*, followed by *Solanum tuberosum*, *Vitis vinifera*, *Solanum lycopersicum*, and other crop species (Figure S3). Further, 15,971 (71.29%) of the total transcripts showed significant similarity with the Swiss-Prot database (Table S1) and 12,908 (57.61%) with the InterProScan database (Table S2).

### 2.3. Gene Ontology (GO) Annotation

**Figure 1:**
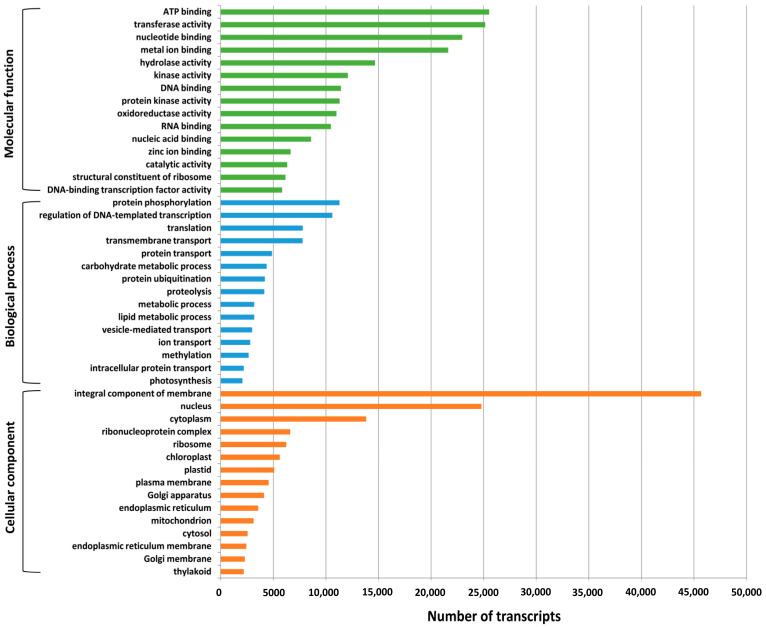
Functional classification of *A. paniculata* transcripts based on GO terms, distributed in three main categories: biological process, molecular function, and cellular component.

We further assigned Gene Ontology (GO) terms to *A. paniculata* transcripts based on sequence homology. A total of 928,242 GO terms were assigned to 17,623 transcripts, and their distribution could be broadly categorized into three major categories: molecular function (414,189, 44.62%), biological processes (270,996, 29.19%), and cellular component (243,057, 26.18%) (Table S3). Based on GO terms, the maximum number of transcripts were present in “ATP binding” and “transferase activity” under the molecular function category; “protein phosphorylation” and “regulation of DNA-templated transcription” under the biological process category; and “integral component of membrane” and “nucleus” under the cellular component category (Figure 1). The transcripts associated with similar functions were assigned to the same GO functional group.

### 2.4. COG Analysis

**Figure 2:**
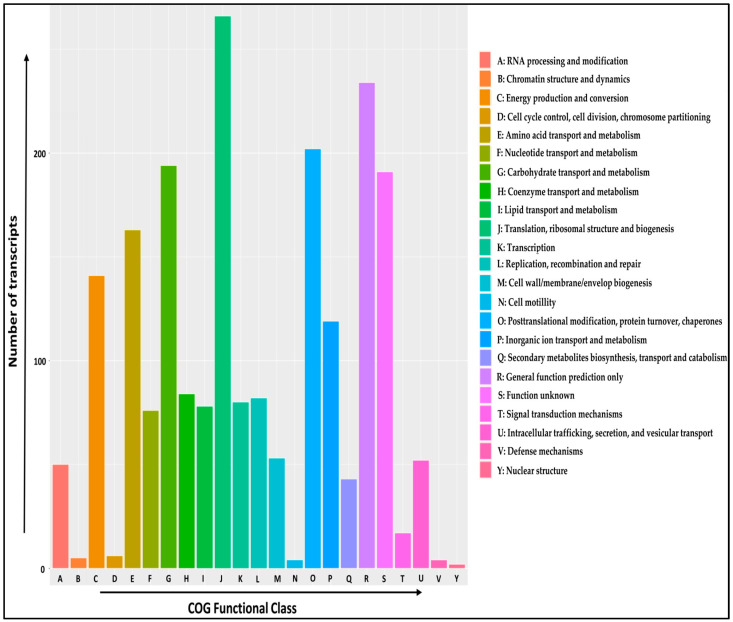
COG functional classification of *A. paniculata* transcripts. The *x*-axis shows the name of the COG functional class, and the *y*-axis shows the number of transcripts.

The COG classification of *A. paniculata* transcripts into different functional clusters of orthologous groups (COG) was based on BLASTX sequence similarity against the COG database with an e-value cut-off of 10^−5^. Out of the total 22,402 transcripts, 2146 were assigned to the 23 COG categories. The largest number of transcripts (266, 12.39%) belongs to the categories of translation, ribosomal structure, and biogenesis, followed by general function prediction only (234, 10.9%), posttranslational modification, protein turnover, chaperones (202, 9.41%), carbohydrate transport and metabolism (194, 9.04%), and amino acid transport and metabolism (163, 7.59%). The least number of transcripts (2, 0.09%) belongs to the category of nuclear structure, while no transcripts were identified for the category’s extracellular structures, mobilome: prophages, transposons, and cytoskeleton (Figure 2).

### 2.5. Metabolic Pathway Enrichment Analysis

**Figure 3:**
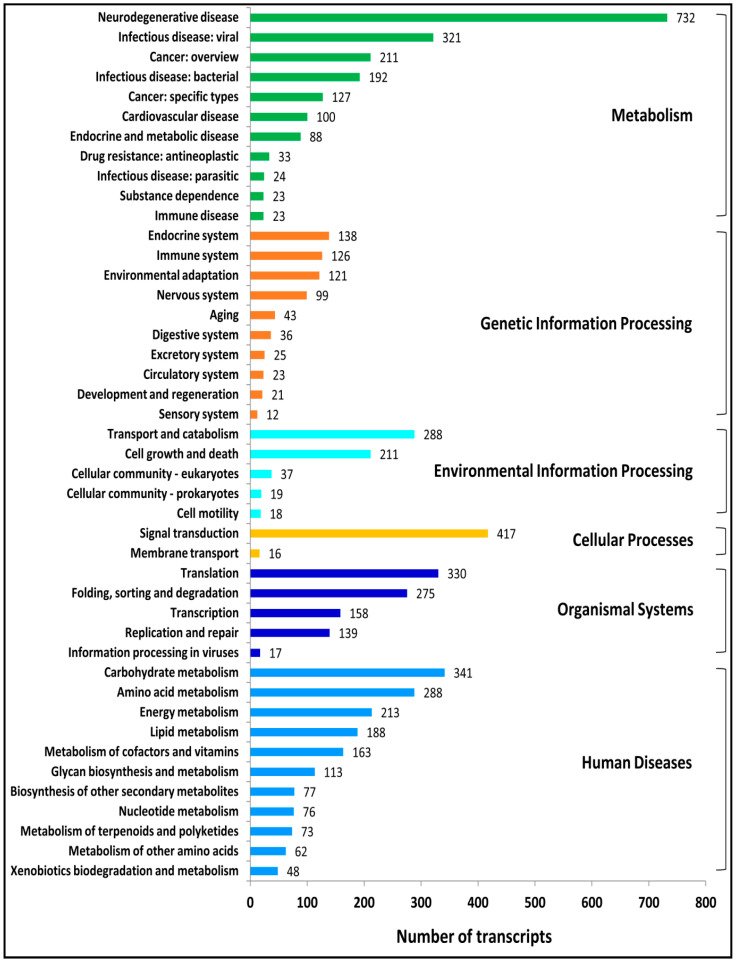
KEGG metabolic pathway mapping of *A. paniculata* transcripts. The *y*-axis shows the name of the KEGG metabolic pathway, and the *x*-axis shows the number of transcripts.

**Figure 4:**
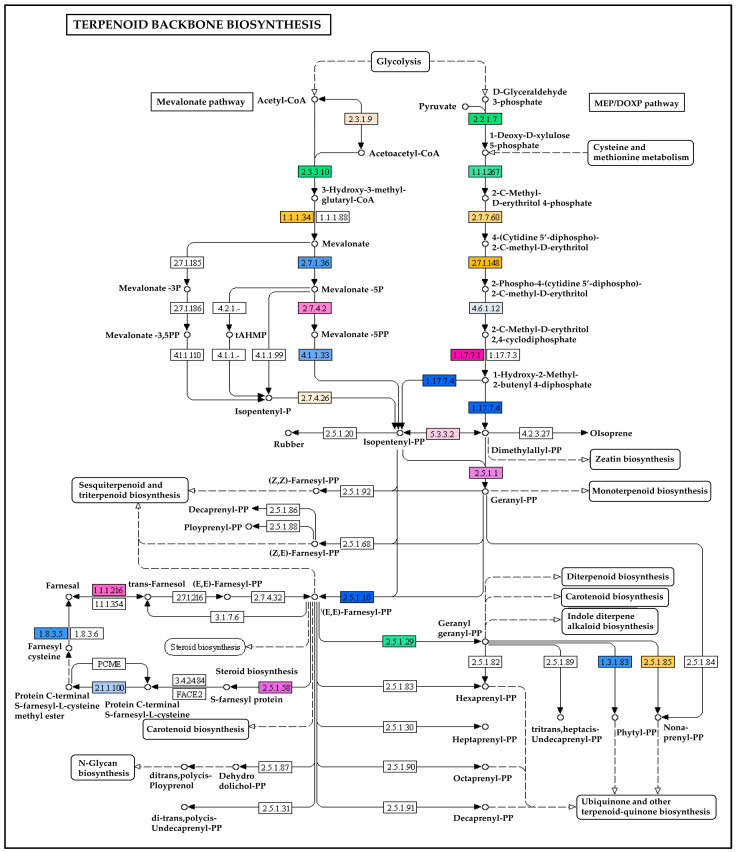
KEGG pathway shows transcripts/enzymes involved in the terpenoid backbone biosynthesis in *A. paniculata* (Kalmegh). Enzymes with enzyme commission numbers are highlighted.

A total of 6088 transcripts had significant matches with the KEGG pathway database and were classified into six categories, including 399 KEGG pathways. Out of the six categories, the largest was metabolism, comprised of 1874 (30.68%) transcripts, followed by human disease (1643; 26.99%), organismal systems (919; 15.10%), genetic information processing (644; 10.58%), environmental information processing (574; 9.43%), and cellular processes (434; 7.13%) (Figure 3; Table S4). Enzyme codes as well as enzyme names were assigned to 2743 transcripts. Out of these, the maximum number of transcripts encodes for transferases, followed by hydrolases, oxidoreductases, lyases, and isomerases (Figure S4). The transcripts that are associated with the terpenoid biosynthesis pathways in KEGG are important. The KEGG pathway analysis of the *A. paniculata* transcripts has shown a total of 32 transcripts mapped to terpenoid backbone biosynthesis (Figure 4), followed by ubiquinone and another terpenoid-quinone biosynthesis (22 transcripts), sesquiterpenoid and triterpenoid biosynthesis (4 transcripts), diterpenoid biosynthesis (3 transcripts), and monoterpenoid biosynthesis (1 transcript).

### 2.6. Transcripts Encoding for the Enzymes Involved in Terpenoids Biosynthesis

A total of 102 different transcripts coding for enzymes involved in the biosynthesis of terpenoids were found in the leaf transcriptome of *A. paniculata*. These enzymes showed a broad range of variability ranging from 53 to 766 amino acids and molecular weight of 5693.51 to 85212.62 kDa. They also showed a wide range of isoelectric points, ranging from basic (4.1 pKa) to acidic (11.83 pKa). As evident from previous reports, the biosynthesis of andrographolide mainly occurs through the mevalonic acid (MVA) and 2C methyl-d-erythritol 4-phosphate/1-deoxy-d-xylulose-5-phosphate (MEP/DXP) pathways [16,17]. It was observed that a greater number of transcripts mapped to the MEP pathway as compared to the MVA pathway in andrographolide biosynthesis. In the MVA pathways, transcripts were found coding for acetyl-CoA C-acetyl transferase (EC2.3.1.9), hydroxymethylglutaryl-CoA reductase (NADPH) (EC1.1.1.34), hydroxymethylglutaryl-CoA synthase (EC2.3.3.10), mevalonate kinase (EC2.7.1.36), phosphomevalonate kinase (EC2.7.4.2), and diphosphomevalonate decarboxylase (EC4.1.1.33), while transcripts coding for 1-deoxy-D-xylulose-5-phosphate synthase (EC2.2.1.7), 1-deoxy-D-xylulose-5-phosphate reductoisomerase (EC1.1.1.267), 2-C-methyl-D-erythritol 4-phosphate cytidylyltransferase (EC2.7.7.60), 4-(cytidine 5’-diphospho)-2-C-methyl-D-erythritol kinase (EC2.7.1.148), 2-C-methyl-D-erythritol 2,4-cyclodiphosphate synthase (EC4.6.1.12), (E)-4-hydroxy-3-methyl but-2-enyl-diphosphate synthase (ferredoxin) (EC1.17.7.1), and 4-hydroxy-3-methylbut-2-en-1-yl diphosphate reductase (EC1.17.7.4) belonged to MEP pathways (Table S5).

A total of 11 transcripts were found to be involved in the MVA pathway (Table S5), including 1 AACT gene, 1 HMGS gene, 4 HMGR genes, 3 MVK genes, and 2 PMK genes. HMGS catalyzes the conversion of Acetoacetyl-CoA to 3-Hydroxy-3-methylglutaryl-CoA, and 3-Hydroxy-3-methylglutaryl-CoA is further converted into mevalonate by HMGR, which is the rate-limiting step in MVA pathway as evidenced from the in silico structural and functional study of HMGR enzyme (ApHMGR) in *A. paniculata* [52]. In the MEP pathway of *A. paniculata,* six transcripts encoded five enzymes, including 1 DXS gene, 1 DXR gene, 1 CMK gene, 1 MDS gene, and 2 HDR genes (Table S5). The first enzymatic reaction in the MEP pathway is carried out by DXSs, which converts pyruvate and D-Glyceraldehyde 3-phosphate into 1-Deoxy-D-xylulose 5-phosphate. Previous studies have confirmed the crucial regulatory and rate-limiting functions of DXS in the biosynthesis of plastidial isoprenoids [53].

### 2.7. Transcription Factor (TF) Analysis

**Figure 5:**
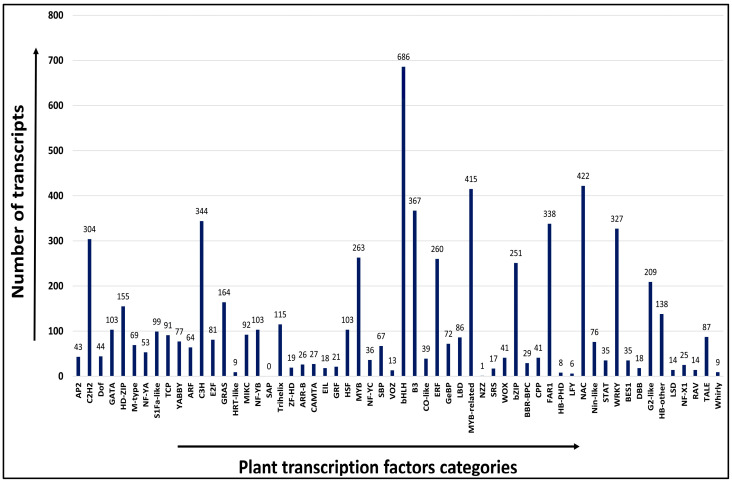
Distribution of TF categories predicted in *A. paniculata*. The *x*-axis shows the name of plant TF categories and the *y*-axis shows the number of transcripts in that TF category.

Transcription factors control the patterns of gene expression, which in turn regulate several biological processes. Out of the total 22,402 transcripts, 6669 showed significant similarity with their corresponding orthologs and were categorized into 57 transcription factor (TF) families. Some plant-specific transcription factors such as bHLH, MYB, NAC, and WRKY have been studied in detail. Out of the total 6669 significant matches, the maximum number of transcripts were categorized into bHLH (686, 3.06%), followed by NAC (422, 1.88%), MYB-related (415, 1.85%), B3 (367, 1.63%), and WRKY (327, 1.45%) families of transcription factors (Figure 5). The bHLH, identified as the largest TF family, showed maximum similarity with *Sesamum indicum* (53 TFs), followed by *Malus domestica* (50 TFs), while the NAC family showed maximum similarity with *Malus domestica* (42 TFs) followed by *Gossypium hirsutum* (32 TFs). Similarly, the MYB-related TF family showed maximum similarity with *Actinidia chinensis* (37 TFs), followed by *Sesamum indicum* (36 TFs), while the WRKY family showed maximum similarity with *Ananas comosus* (35 TFs), followed by *Sesamum indicum* (28 TFs) (Table S6). The annotated transcripts were analyzed in silico to predict the TF gene families involved in the synthesis of biochemical compounds having medicinal values, such as cytochrome p450, protein kinases, heat shock proteins (HSPs), transporters, etc. A total of 106, 766, 26, and 406 transcripts belong to cytochrome p450, protein kinases, heat shock proteins, and transporter categories, respectively (Table S7).

### 2.8. Validation of Transcription Factors

A total of 15 TF genes from three different TF families, including bHLH (bHLH14, bHLH15, bHLH53, bHLH82, bHLH98), MYB (MYB113, MYB186, MYB228, MYB733, MYB397), and NAC (NAC38, NAC150, NAC151, NAC159, NAC191) were validated by PCR amplification on two *A. paniculata* accessions (IC-111287 and IC-342136). All primers showed amplification of transcription factor genes on the cDNA of both *A. paniculata* accessions (Figure S5), which supports the quality of our transcriptome assembly. Among the bHLH TF family genes, all five genes specific primers gave proper amplification except primer 3 of gene bHLH15; in the MYB family genes, all five genes specific primers amplified properly except primer 3 of gene MYB186; and in the NAC TF gene family genes, all five genes specific primers gave proper amplification in both the accessions (IC-111287 and IC-342136) except primer 2 of gene NAC 150 and primer 1 of gene NAC191.

### 2.9. Frequency and Distribution of EST-SSRs

**Figure 6:**
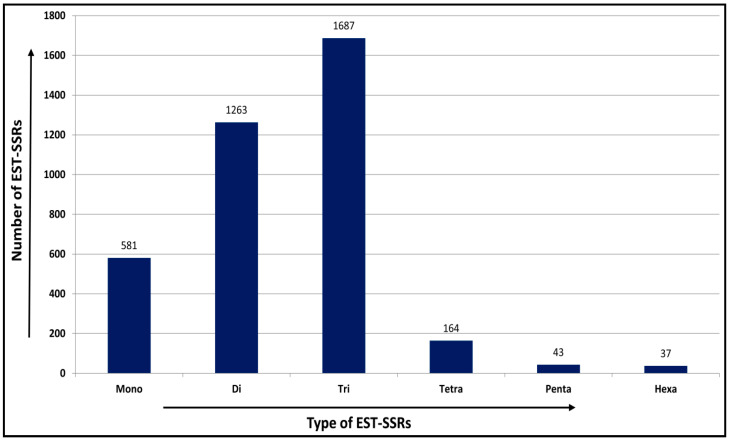
Frequency and distribution of EST-SSR types predicted in *A. paniculata.* The *x*-axis shows the types of EST-SSRs, and the *y*-axis shows the frequency of predicted EST-SSRs.

A total of 4254 EST-SSRs were identified from 3661 transcripts, representing 16.34% of the total (22,402) transcripts. Out of 4254 EST-SSRs, 480 were compound repeats motifs, 580 were mono-nucleotide and complex repeat motifs that were removed, and 3194 perfect EST-SSRs (di- to hexa-repeat motifs) representing 2839 transcripts were obtained. Among the 2839 transcripts, 2517 transcripts contained a single EST-SSR locus, whereas the remaining 322 transcripts contained two or more EST-SSR loci. The tri-nucleotide motif was the most common repeat unit with a frequency of (1687, 39.65%), followed by di- (1263, 29.68%), tetra- (164, 3.85%), penta- (43, 1.01%) and hexanucleotide repeats (37, 0.86%) (Figure 6). The frequency of EST-SSRs and the number of repeats showed an inverse relationship (Table S8). The five tandem repeats EST-SSR (1090, 28.88%) were the most abundant, followed by six tandem repeats (810, 21.46%), seven tandem repeats (434, 11.50%), whereas tandem repeats from eleven to thirty-five each account for less than 5% of the EST-SSRs (Table S8). The EST-SSR tandem repeats were 10 to 317 bp in length, and 15 bp was the most frequently observed length (942, 22.14%), followed by 18 bp (589, 13.84%), 12 bp (411, 9.66%), and 14 bp (275, 6.46%). In the di-nucleotide repeat units, “AT” was the most abundant with a frequency of 12.67%, and among the tri-, tetra-, penta-, and hexanucleotide repeat motifs, “CCG,” “ATAC”, AAAAT”, and “CCACCG” were the most abundant with frequencies of 2.54%, 0.16%, 0.08%, and 0.05%, respectively (Table S9).

### 2.10. Assessment of Genetic Diversity in A. paniculata

**Figure 7:**
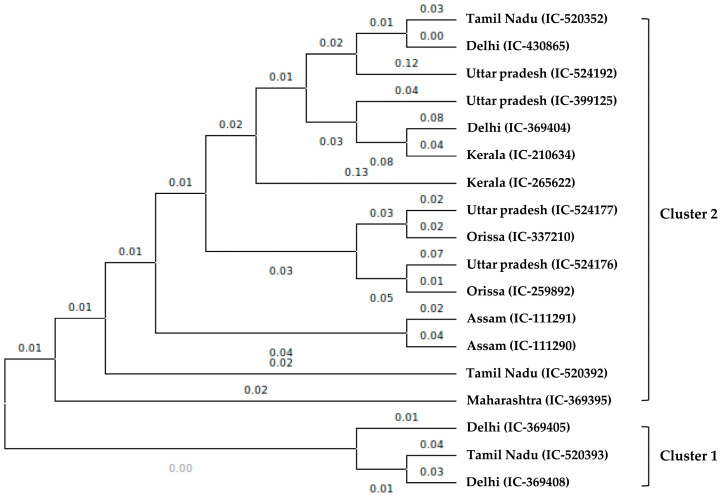
Dendrogram of 18 accessions based on the 20 polymorphic EST-SSR markers using the UPGMA method of cluster analysis.

Eighteen *A. paniculata* accessions collected from different states of India, such as Assam, Delhi, Kerala, Maharashtra, Orissa, Tamil Nadu, and Uttar Pradesh, (Figure S6) were used to validate de novo-designed EST-SSRs generated from our EST dataset. A total of 53 primer pairs were randomly selected and synthesized for the assessment of the genetic diversity study among the 18 accessions of *A. paniculata*. Out of the 53 selected primer pairs, 39 were successfully amplified by the *A. paniculata* genomic DNA, while the remaining primer pairs failed to amplify. From the 39 amplified EST-SSRs, 20 primer pairs showed polymorphic bands (Figure S7) which produced a total of 41 alleles. The overall percentage of polymorphism observed was 68.33%. The number of alleles amplified per locus ranged from 2 to 3, with an average of 2.05 alleles per locus (Table S10) in the case of polymorphic markers. The observed heterozygosity (Ho) ranged from 0.0 to 1.00 with an average of 0.292, and expected heterozygosity (He) ranged from 0.10 to 0.66 with an average of 0.310, respectively. The PIC value for polymorphic markers ranged from 0.59 to 0.099 with a maximum PIC value with primer-34 and minimum PIC value with primer 6 (Table S10). The UPGMA cluster analysis based on 41 alleles amplified revealed two distinct sub-clusters (Figure 7), in which all 18 accessions were distributed. The accessions collected from seven different states of India were not showing any geographical isolation and accessions from Delhi (IC-369405 and IC-369408) were grouped with accession from Tamil Nādu (IC-520393) in cluster 1. Since these EST-SSR markers separated closely related accessions, these markers can be very useful for the study of genetic diversity studies among Andrographis accessions and their application in core development and conservation.

### 2.11. Meta-Transcriptome Assembly and Data Analysis

A total of 137,725 and 12,898 transcripts have been generated from the Illumina as well as Ion-torrent sequencing platforms datasets were merged with our in-house de novo assembled transcripts (22,402) to generate a non-redundant meta-transcriptome assembly (98,514 transcripts) using the CD-HIT program with 90% identity. By meta-transcriptome assembly, we have generated an improved transcriptome assembly of 98,514 transcripts with an N50 value of 1902 bp and an average transcript length of 1175 bp, which is better than earlier reported transcriptome assemblies by Garg et al. [15], Cherukupalli et al. [16], Li et al. [17], Patel et al. [8], and Sharma et al. [18] in *A. paniculata*. Cherukupalli et al. [16] reported an N50 value of 1880 and an average transcript length 1061. This meta-transcriptome assembly was further used for the identification of EST SSRs, transcription factors, and the development of a genomic resource database. 

### 2.12. Database Resource of A. paniculata

A database named ApTransDB was developed to provide uninterrupted public access and deliver useful information about the genomic resources of an important medicinal plant, *A. paniculata*. The database contains a total of 37,814 perfect SSR, 1462 compound SSR, and 170,017 imperfect SSR markers with their three pairs of primers information, 9258 transcription factors, 60,662 annotated transcripts, and important gene families such as cytochrome P450, protein kinases, heat shock proteins, and transporters related to biochemical compounds biosynthesis in *A. paniculata*. The number of perfect SSR markers, compound SSR, imperfect SSR, and transcription factors has increased in the database due to meta-transcriptome analysis done using other transcriptome resources available publicly. This database can be accessed using various search options, and users can extract useful information as well as download various structural (SSR details) and functional (annotation details) features of the *A. paniculata* transcriptome. ApTransDB has nine different tabs with six sub-tabs under the Database tab (About, Database Search, BLAST Search, Tutorials, Downloads, Feedback, Links, Team, and Contact Us). SSR search can be performed in the Perfect SSR Search sub-tab using different search criteria such as SSR Type, SSR Id, SSR motif, Motif repeat and SSR length, and Annotation keyword. Similarly, details about different TF categories present in *A. paniculata* can be accessed via the Get TF Categories sub-tab, gene family details via the Gene Families sub-tab, and details of annotated transcripts via the Get GO Categories sub-tabs. A Local BLAST feature is also incorporated in ApTransDB, which enables the user to compare their query sequence with the *A. paniculata* transcripts. ApTransDB can be accessed freely via the web address http://www.nbpgr.ernet.in:8080/Andrographis/ (accessed on 6 November 2022).

## 3. Discussion

Next-generation sequencing (NGS) has been efficiently used to analyze the transcriptome sequencing and assembly of many model and non-model plant species because of its high efficiency, accuracy, and affordability. *Andrographis paniculata* is widely distributed in India and has high therapeutic value. However, as a valuable species, the available genomic information is still limited for the exploitation of this plant species. In the present study, an effort has been made to generate genomic resources through the transcriptome of leaves of *A. paniculata*. A total of 22,402 assembled transcripts were generated with an N50 and average transcripts lengths of 1007 bp and 884 bp, respectively. The numbers of assembled transcripts were less than those of earlier published reports by Garg et al. [15] (69,011), Cherukupalli et al. [16] (83,800), Li et al. [17] (43,683), Patel et al. [8] (38,292), and greater than those of Sharma et al. [18] (20,498) for the same species. The N50 was 1007 bp in our analysis (Table 1), which is comparable with the de novo transcriptome assembly of *S. japonicas* (846 bp) [54], *Rhododendron rex* Lévl. (752 bp) [19], and *Zanthoxylum bungeanum* (846 bp) [55], thereby indicating the quality of the de novo assembled transcriptome of *A. paniculata* in the present study.

To predict the biological function of assembled transcripts, functional annotation was performed using different public databases, such as the NCBI-Nr database, Swiss-Prot, InterProScan, KEGG, COG, and GO databases. These annotation details can provide valuable information about genetic diversity and chemotypes present in *A. paniculata*. In our study, a total of 19,264 (86%) transcripts were successfully annotated, which was higher than that of *Curcuma alismatifolia* (78.6%) [56], and *Centella asiatica* (53.04%) [57]. In addition, a total of 3138 (14%) transcripts cannot match any functional annotations in public databases, possibly due to the short sequence length discrepancies or limited annotation of the genus *Andrographis* and related species in the current database. The frequency of transcripts greater than 500 bp within the assembled transcripts showed a higher percentage of transcripts annotation. Many genomes are classified into a broad range of GO categories, and COG classifications revealed that assembled transcripts in the *A. paniculata* genome represent a wide range of transcripts [19,58]. Of the three GO categories, most of the transcripts from the present study belong to the molecular function category, followed by biological process, and cellular component (Table S3), which were consistent with the report for *Menispermum* L. (moonseed) [59]. Overall, 2146 transcripts were assigned to the 23 COG functional categories; a similar observation was reported on *Rhododendron rex* [19]. Moreover, 6088 (27.17%) transcripts had significant matches with the KEGG database and were assigned to six main categories, including 399 KEGG pathways.

In the present analysis, a total of 6669 transcripts were categorized into 57 TF families. Of the 57 categories, most transcripts were categorized into bHLH, followed by NAC, MYB-related, and WRKY TF gene families (Figure 5); a similar distribution was reported in *Tinospora cordifolia* [50]. The bHLH family was the most abundant in our analysis. Being the largest TF group in plants, the bHLH family demonstrated its essential role in responses to abiotic stresses, such as drought, salinity, and chilling [60]. The WRKY TF are among the largest families of transcriptional regulators mainly involved in plant growth and developmental processes [61] and the NAC family of TFs is involved in the regulation of biotic and abiotic stress responses [62]. The PCR amplification of TF gene-specific primers from three important TF families, i.e., bHLH, MYB, and NAC, in two different *A. paniculata* accessions, as well as molecular cloning and characterization of the NAC86 gene by Ramesh et al. [63], showed the quality of our transcriptome assembly. A total of 106 transcripts have been identified as being related to the cytochrome p450 enzyme group, and 766 transcripts are related to protein kinases; a similar type of transcript has already been reported in *T. cordifolia* [64], an important medicinal plant that also produces secondary metabolites using terpenoid pathway. Similarly, 26 transcripts related to HSPs were found and are known to function as molecular chaperones, which are involved in the treatment of many diseases, and 406 transcripts related to transporter genes were found, out of which 89 were ATP-binding cassette (ABC transporters). The ATP-binding cassette (ABC transporters) are one of the largest transport systems super families, mainly involved in the transport of plant secondary metabolites such as alkaloids, terpenoids, polyphenols, and quinones [65].

In comparison to dominant markers, simple sequence repeats (SSRs) or microsatellite markers (codominant markers) are considered more important for genetic diversity assessment and offer outstanding benefits because of their low cost and user-friendly nature. SSRs fall into two categories: genomic SSRs, which are derived from randomly selected non-expressed genomic regions, and EST-SSRs, which are derived from gene-rich regions. Generations of genomic SSRs are relatively time-consuming, expensive, and labor-intensive as compared to EST-SSRs. Furthermore, genomic SSRs have neither genic function nor close linkage to transcriptional regions. The EST-SSR markers are very important genomic tools because of their close association with functional genes that regulate some important economic and agronomic characteristics [50]. Additionally, EST-SSR markers also have higher transferability to related species because they are derived from expressed genes, which are likely to be more conserved than the SSRs developed from non-genic regions [54,66]. In this study, a total of 4254 possible EST–SSRs were identified from 3661 transcripts, which represents 16.34% of the total (22,402) transcripts. Tri-nucleotide (39.65%) repeats were the most abundant type, which is consistent with previous studies on moon seed [59] and *C. alismatifolia* [56], followed by di- (29.68%), tetra- (3.85%), penta- (1.01%), and hexa-nucleotide (0.86%) repeats. Tri-nucleotide repeats are the most abundant SSR motifs because open reading frames do not induce insertions and deletions within translated regions, whereas frameshift mutations may restrict the development of other motif types [67]. In addition, regarding the mononucleotide repeats, as in most plants (*Curcuma alismatifolia* and *Panicum miliaceum* L.), A/T repeats were far more abundant than G/C repeats [55,56,58]. In the tri-nucleotide repeats, the most dominant repeat motif was CCG (2.54%) which is similar to our earlier report on *T. cordifolia* [64], followed by CGG (2.31%), and the GAA motif (1.91%). Interestingly, there were CCG/CGG tri-nucleotide repeat motifs in *A. paniculata*, which are typically most prevalent in monocots. However, our results support the rarity of occurrence of CCG/CGG repeat units in a large number of dicotyledonous plants (*C. sinensis*, *Coffea canephora*, *M. truncatula*, *G. max*, etc.) due to high GC content and consequent codon usage bias in monocots [66,68].

In this study, 53 primer pairs were designed, with 39 primer pairs effectively producing clear amplicons. Among the eighteen genotypes, 20 (37.7%) polymorphic genic SSR markers were identified. This is higher than the number reported in *Elymus sibiricus* (22.4%) [69], and *Juglans mandshurica* Maxim. (30.8%) [70], and less than *Rhododendron rex* Lévl. (63%) [19] and *Paeonia suffruticosa* (39.9%) [71]. Moreover, 14 primer pairs failed to produce amplification, which may be due to the large introns, and chimeric primers [72]. We detected 41 polymorphic alleles in 20 loci among 18 *A. paniculata* accessions based on the 20 polymorphic EST–SSR markers. The number of alleles per locus ranged from 2 to 3 with a mean of 2.05 alleles, and the average major allele frequency was 0.77 which is comparable with *T. cordifolia* [50]. The averages of observed heterozygosity (Ho) and expected heterozygosity (He) were 0.292 and 0.310, respectively, which is similar to the *Rhododendron rex* Lévl (Ho = 0.323, He = 0.372) [19] and lower than that of *Menispermum* populations (Ho = 0.38, He = 0.58) [59]. The PIC value of each EST-SSR reflects the allelic diversity and frequency of the sampled individuals, and was used to evaluate the level of genetic information [71]. In our analysis, the PIC values ranged from 0.099 and 0.592, with an average of 0.258, which represents a moderate level and is consistent with the PIC values observed in *Rhododendron rex* Lévl, which ranged from 0.154 to 0.870, with an average of 0.482 [19]. Thus, the newly developed EST–SSR markers can be an efficient tool for genetic analysis and evolutionary study among a wide range of diverse *A. paniculata* populations.

In addition, we have developed a user-friendly web resource for public access. Our database features such as EST-SSR markers and their three pairs of primers, annotated transcripts categorized different sub-categories, transcription factors, and important gene families related to the biosynthesis of secondary metabolites share similarities to the *T. cordifolia* database (TinoTranscriptsDB) [64]. Some other databases, such as the MusatransSSRDB [47] and Vigna mungo transcriptome database (VmTDB) [48], also contain EST-SSR markers and transcription factors information similar to the present database. In the present database, users can extract the information as per their requirements based on unique EST-SSR IDs, transcript IDs, domain search, GO IDs, SNP IDs, and TF category, as well as the name of gene families. The presence of important gene families related to the biosynthesis of secondary metabolites such as cytochrome-p450, protein kinases, HSPs, and transporters is a unique feature of our genomic resource database, which is present in *T. cordifolia* genomic resources and lacking in other genomic resources, such as the MusatransSSRDB [47], VmTDB, and CTDB genomic resource databases [51].

## 4. Materials and Methods

### 4.1. Plant Materials and RNA Isolation

Young leaves of disease-free *A. paniculata* plants were collected from an experimental farm (located at Issapur) of the National Bureau of Plant Genetic Resources, New Delhi, India. After harvesting, leaves were immediately fixed in liquid nitrogen, and stored at −80 °C until the DNA was isolated. For the cDNA library preparation, *A. paniculata* accession IC-111287 was selected based on its morphological and biochemical considerations. Frozen leaf tissues were ground to a fine powder using liquid nitrogen to maintain the RNA integrity. Total RNA was isolated according to the instruction of the TRIzol^®^ kit (Life Technologies Invitrogen, Carlsbad, CA, USA) and purified using an mRNA purification kit (Promega, Madison, WI, USA) to exclude the tRNA and rRNA and enrich the mRNA quality.

### 4.2. cDNA Library Preparation and Sequencing

For the cDNA library preparation enriched mRNAs were reverse-transcribed by Powerscript™ II (Takara, Tokyo, Japan) with PCR primers SMART IV™ Oligonucleotide (5′-AAGCAGTGGTATINOSPORAAACGCAGAGTGGCCATTACGGCCGGG-3′) and CDS III/3′ PCR Primer (5′-ATTINOSPORATAGAGGCCGAGGCGGCCGAC ATG-d (T) 30N-1N-3′) (Clonetech, Mountain View, CA, USA). Next, for double-strand cDNA amplification, long-range PCR was performed using the LA Taq enzyme (Takara, Japan) for 25 cycles (95 °C for 30 s, 68 °C for 8 min) according to the SMART™ cDNA Library Construction Kit. Finally, to produce high-quality cDNAs, double-stranded cDNAs were purified using a DNA purification kit (Qiagen, Hilden, Germany). About 10 μg of cDNA were used for a half-plate sequencing run using 454 GS-FLX pyrosequencing technology. Before the sequencing, full-length cDNA was sheared stochastically to randomly represent all transcripts. A total of 748,409 raw sequence reads were generated and deposited at NCBI Short Read Archive (SRA) under the accession number SRP018666. The assembled transcripts were also deposited in the TSA (transcriptome shotgun assembly) database of NCBI with the accession number GBSY00000000.

### 4.3. Reads Quality Assessment and De Novo Transcriptome Assembly

**Figure 8:**
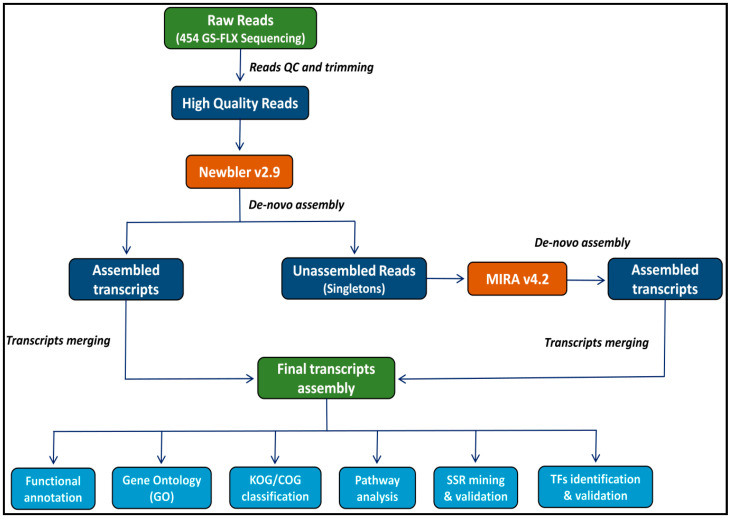
Process flow diagram of *Andrographis paniculata* transcriptome assembly and analysis.

The raw reads were quality checked and filtered out to remove low-quality reads as well as reads containing adapter sequences. Reads shorter than 60 bp were removed before performing the assembly. The resultant high-quality filtered reads were de novo assembled using the GS de novo assembler Newbler v2.9 (Roche, IN, USA) with the assembly parameters of a minimum overlap length of 40 bp and minimum overlap identity of 90%. The remaining unassembled reads (singletons) were extracted from the Newbler-trimmed 454 reads file using Seqtk software [73]. The CLC Genomics Workbench (http://www.clcbio.com (accessed on 12 August 2022)) was used to map back singletons against Newbler assembled transcripts, and unmapped singletons were eliminated as duplicates. The remaining reads were assembled using MIRA v4.2 with the following parameters: est, accurate, de-novo, 454. To remove duplicates, the singletons that failed to assemble by MIRA v4.2 were extracted and mapped back to the MIRA assembled transcripts using CLC Genomics Workbench. Further, transcripts less than 200 bps generated by MIRA v4.2 were removed, and the remaining transcripts were merged with the transcripts generated by Newbler v2.9 to get the final transcriptome assembly. The graphical representation of the detailed methodology followed is shown in Figure 8.

### 4.4. Functional Annotation and Biological Pathways Assignment

The functional annotation of final non-redundant transcripts was carried out using BLASTX searches against various databases, including NCBI non-redundant (NR) protein database, Swiss-Prot, Kyoto Encyclopedia of Genes and Genomes (KEGG), Gene Ontology (GO), Protein family (Pfam), and COG databases, with a cut-off E-value of 10^−5^. Functional descriptions were assigned to non-redundant transcripts with BLAST results against the NCBI NR and Swiss-Prot protein databases using Blast2GO v6.0.3 [74]. Blast2GO was also used to perform gene ontology (GO) enrichment, pathway analyses (using KEGG), and enzyme commission (EC) number assignment. Gene ontology annotation categorized the functionally annotated transcripts into three GO categories, namely Biological Process (BP), Cellular Components (CC), and Molecular Functions (MF). Pathway mapping and gene orthologous assignment of the annotated transcripts were done using **K**EGG **A**utomatic **A**nnotation **S**erver (KASS) [75] with default parameters. The KASS gives functional annotation of genes by BLAST search against the manually curated KEGG GENES database. Based on the similarity with the KEGG database, annotated transcripts were assigned unique enzyme commission (EC) numbers. Further, transcripts under the respective EC numbers were used to map them to the KEGG biochemical pathways. Furthermore, functional annotation was also performed using the COG database (http://www.ncbi.nlm.nih.gov/COG/ (accessed on 22 August 2022)) based on the COG information obtained from the string.

### 4.5. Identification of Transcription Factors and Gene Families

Identification of transcription factor families was done by using BLASTX similarity search of all annotated transcripts against the protein sequences downloaded from the Plant Transcription Factor Database (PlantTFDB v5.0; http://planttfdb.gao-lab.org/ (accessed on 24 August 2022)) with the parameters bit score > 100, and E-value cut-off of 10^−6^. Based on the annotated transcripts, important gene families related to the biosynthesis of secondary metabolites in *A. paniculata* were also analyzed.

### 4.6. Selection of TF Genes, Primer Designing, and PCR Amplification

A set of three important plant-specific TF families, i.e., NAC, MYB, and bHLH, were selected for their transcript validation. The selection of these TF families was done based on the presence of the maximum number of TFs in these TF families as well as the importance of these TF families in the plant defense system. The nucleotide sequences of 15 TFs, five from each family, were carefully analyzed by comparing the members of the same family for similarity and distinction and then targeted to design the PCR primer pairs. The primer pairs specific to all 15 TF genes were designed using Primer3 v1.0 software (https://github.com/primer3-org/primer3/ (accessed on 26 August 2022)) with the following parameters: primer lengths of 20–25 bp, the size of PCR product of 100–250 bp, annealing temperature of 60 ± 5 °C, and GC content of 40–60%. The list of TFs gene primer sequences along with their TF ID is given in Table S11.

The leaves from two *A. paniculata* accessions (IC-111287 and IC-342136) were collected after 60 days of sowing and stored in liquid nitrogen immediately after harvest. Total RNA isolation using an RNA isolation kit (Thermo Fisher Scientific, Waltham, MA, USA) was done as per manufacturer protocol. The quality and quantity of purified RNA were checked using Nanodrop. The cDNA was synthesized with the help of a cDNA Synthesis kit (Invitrogen, Waltham, MA, USA) and confirmed by PCR amplification.

Final PCR amplification was performed in a total reaction volume of 25 µL containing 2.5 µL 10× PCR buffer, 1 µL dNTP (10 mM), 1 µL MgCl_2_ (2.5 mM), 0.5 Taq polymerase (Thermo Scientific, Waltham, MA, USA), 1 µL of each primer (10 mM), and 19 µL autoclaved distilled water. PCR amplification was performed in a thermocycler (G storm, Essex, England) using the following conditions: initial denaturation at 94 °C for 5 min, followed by 35 PCR cycles of denaturation at 94 °C for the 30 s, annealing temperature at 58 °C for 30 s, extension at 72 °C for 1 min, and a final extension at 72 °C for 10 min. The PCR amplicons were checked on a 1.5% agarose gel (Lonza, Rockland, ME, USA) for 2 h at a constant supply of 100 V. Gel images were captured under UV with the help of a gel documentation system (Alpha Imager^®^, Bengaluru, Karnataka, India).

### 4.7. Identification of EST-SSR Markers and Primer Designing

Simple sequence repeats were identified from the final non-redundant transcripts set using the MIcroSAtellite identification tool v1.0 (MISA) (http://pgrc.ipk-gatersleben.de/misa/ (accessed on 25 August 2022)). The minimum SSR length criteria were defined as six iterations for di- and five iterations for tri-, tetra-, penta-, and hexa-SSR repeat units. A maximum of 100 interrupting bases were allowed between two SSRs in a compound microsatellite. For this study, SSR loci containing repeat units of 2–6 nucleotides were considered. Mononucleotide repeat and complex SSR types were excluded from the study. The primer pairs were designed from the flanking regions of SSR motifs using Primer3 v1.0 (https://github.com/primer3-org/primer3/ (accessed on 26 August 2022)) [76] with the following parameters: primer length = 20–25 bp, size of PCR product = 100–250 bp, with an optimum of 280 bp, annealing temperature = 65 °C, and GC content of 40–60% with an optimum of 50%. The list of EST-SSR primer pairs is presented in Table S12.

### 4.8. Assessment of EST-SSR Polymorphism

A total of 18 accessions of *A. paniculata* were selected for the genetic diversity analysis using EST-SSR markers (Table S13). These 18 accessions were collected from seven different states of India (Assam (IC-111290, IC-111291), Delhi (IC-369404, IC-369405, IC-369408, IC-430865), Kerala (IC-265622, IC-210634), Maharashtra (IC-369395), Orissa (IC-337210, IC-259892), Tamil Nadu (IC-520352, IC-520392, IC-520393), and Uttar Pradesh (IC-399125, IC-524176, IC-524177, IC-524192)) (Figure S6). Genomic DNA was extracted from the young leaf tissues using the Cetyl Trimethyl Ammonium Bromide (CTAB) method [77] used for the PCR amplification of EST-SSR markers. Approximately five grams of fresh leaves were crushed into liquid nitrogen using a mortar and pestle. The quality of the resulting DNA was assessed using a 0.8% agarose gel and quantified using Nanodrop (Thermo Fisher, USA). For all eighteen accessions used in the polymorphism study, a working concentration of 10 ng/µL DNA stock was prepared, and PCR conditions were also optimized for the EST-SSRs primers. A total of 53 primer pairs were initially screened to study the polymorphism in *A. paniculata* accessions. Final PCR amplification was performed in a total of 10 μL of reaction volume containing 1× buffer (20 mM Tris-HCl, 50 mM KCl), 1.5 mM MgCl_2_, 0.24 mM of each dNTPs, 0.5 U of Taq polymerase (Promega, USA), 0.8 μm of primer, and 25 ng of template DNA. Each genotype primer combination was amplified twice to check for reproducibility. Only reproducible primers were selected for the genetic diversity study. PCR amplification was performed in a thermal cycler (G-Storm, London, UK) using the following conditions: initial denaturation step at 94 °C for 3 min, followed by 35 cycles of 94 °C for 1 min, annealing temperature ranged from 50 °C to 53 °C for 1 min, and 72 °C for 2 min; the final extension at 72 °C was held for 5 min.

PCR amplified products were subjected to horizontal gel electrophoresis using metaphor agarose gel (4%) in 1× TBE buffer at 80 V for 4 h using Broviga^TM^ standard submarine gel electrophoresis unit. The 100 bp DNA ladder (Fermentas, USA) was used as a standard-size marker. Metaphor agarose gel was stained with 0.5 µg/mL ethidium bromide solution, and gels were visualized and documented under ultraviolet light using Alpha Imager 1200^TM^ (Alpha Innotech Corporation, San Jose, CA, USA).

### 4.9. Genetic Diversity Data Analysis

DNA bands were scored based on their fragment size (bp), and the data matrix was prepared accordingly. POPGENE V1.32 software was used for the calculation of the number of observed alleles (Na), expected heterozygosity (He), and observed heterozygosity (Ho) [78], and the polymorphic information content (PIC) was calculated using the Power Marker v3.25 [79]. The genetic similarities were calculated using the dice similarity coefficient, and the dendrogram was generated using Unweighted Pair Group Method with the Arithmetic Average (UPGMA) method of the NTSYS-pc software [80].

### 4.10. Meta-Transcriptome Assembly for the Generation of a Database Resource

For de novo meta-transcriptome assembly, our in-house leaf tissue sequences RNA-Seq data of *A. paniculata* generated through the Roche-454 GS-FLX platform (SRA Acc. No. SRR719255), and other publicly available RNA-Seq data of the same species and tissue generated through different sequencing platforms including Illumina (SRA Acc. No. SRR1519324, SRR12791806, SRR12791807, SRR1292497) and semiconductor-based technology Ion Torrent (SRA Acc. No. SRR8500525), have been used. The transcriptome shotgun assembly (TSA) of the Roche 454 dataset (22,402 transcripts) was downloaded from the NCBI TSA database, while Illumina SRA reads accession no. SRR1519324, SRR12791806, SRR12791807, and SRR1292497 and Ion Torrent SRA reads accession no. SRR8500525 were downloaded from the NCBI SRA database. Further, raw reads were quality-checked using FASTQC; http://www.bioinformatics.bbsrc.ac.uk/projects/fastqc (accessed on 10 August 2022), and the adapters and poor-quality bases were trimmed using Trimmomatic V3.39. Afterward, filtered reads of Illumina and Ion Torrent platforms were assembled separately using trinity software with default parameters. The Illumina assembled transcripts and Ion Torrent assembled transcripts were further merged with our in-house de novo assembled transcripts to generate a non-redundant meta-transcriptome assembly using the CD-HIT program with 90% identity. This meta-transcriptome assembly was further used for the development of *A. paniculata* Microsatellite Markers and Transcription Factors Database (ApTransDB).

### 4.11. Meta-Transcriptome Analysis

The final meta-transcriptome assembly was used as input in the Krait v1.4.0 software (https://github.com/lmdu/krait, accessed on 26 August 2022) [81] for the identification of microsatellite markers (SSRs) using the following parameters: six repeat units for di-nucleotide and five repeat units for tri-, tetra-, penta- and hexa-nucleotide repeats, and the maximum number of bases interrupting 2 SSRs in a compound microsatellite to be 100 bp. The primer pairs were designed from the 200 bp flanking regions of SSR motifs using Primer3 v1.0 (https://github.com/primer3-org/primer3/, accessed on 26 August 2022) [76] with the following parameters: primer length = 20–25 bp, the size of PCR product = 150–300 bp, with an optimum of 280 bp, annealing temperature = 65 °C, and GC content of 40–60% with an optimum of 50%. The functional annotation of the meta-transcripts was carried out using BLASTX searches against various databases, including NCBI NR, Swiss-Prot, KEGG, GO, Pfam, and COG databases, with a cut-off E-value of 10^−5^. For the identification of transcription factor families, annotated transcripts were BLASTX searched against the PlantTFDB v5.0 protein database (http://planttfdb.gao-lab.org/, accessed on 27 August 2022) with the parameters bit score > 100, and E-value cut-off of 10^−6^.

### 4.12. Database Resource Development

A dedicated database of transcriptome-based EST-SSR markers and TFs along with transcripts annotation information and important gene families related to the biosynthesis of secondary metabolites, such as cytochrome-p450, protein kinases, HSPs, and transporters, was developed for scaling up research by the scientific community involved in the genetic improvement of *A. paniculata*. The Andrographis Transcripts and SSR Database (ApTransDB) is a three-tier-based relational database that contains information on various categories of SSR markers, TFs, and functionally annotated transcripts of *A. paniculata*. It can be used to retrieve SSR information based on user queries of interest along with an important data download option. The interactive user-friendly web interface of the database was designed and developed using a server-side web programming language (ASP.NET), and database tables were stored in MS SQL Server 2019.

## 5. Conclusions

The transcriptome data generated in this study were used in generating genomic resources and their validation. These resources can be subsequently used for the genetic enhancement of economically important traits in *A. paniculata*. The generation of EST-SSRs, designed from annotated and non-annotated ESTs, will serve as an invaluable resource for assessing genetic diversity among *A. paniculata* germplasms and may also assist in correlating the inherent chemo-diversity of germplasm with allelic diversity of functional importance, if any. For any plant species, the extent of available diversity in their germplasm is an invaluable asset for their appropriate utilization. Kumar et al. [63] performed cloning and characterization of the full-length NAC86 TF gene associated with the secondary metabolite biosynthetic pathway using the ApTransDB database. This study shows the possibilities and the utility of this database, which can be further extended for mining transcripts/genes associated with other related pathways.

## Figures and Tables

**Table 1:** Summary statistics of the *A. paniculata* transcriptome assembly analysis.

S. No.	Raw Data	Statistics
1.	No. of raw reads	748,409
2.	Length of raw reads (bp)	305,186,073
3.	No. of trimmed reads	730,670
4.	Length of trimmed reads (bp)	301,674,417
5.	The average length of trimmed reads (bp)	500
	Newbler Assembly	Statistics
6.	No. of transcripts	16,149
7.	Size of assembly (bp)	15,635,774
8.	N50 (bp)	1184
9.	Average transcript length (bp)	968.2
10.	Longest transcript length (bp)	5975
11.	Smallest transcript length (bp)	200
12.	Total no. of singletons (by Newbler)	65,004
	MIRA Assembly	Statistics
13.	No. of transcripts	6253
14.	Size of assembly (bp)	4,186,517
15.	N50 (bp)	650
16.	Average transcript length (bp)	669.5
17.	Longest transcript length (bp)	2067
18.	Smallest transcript length (bp)	297
	Final Assembly	Statistics
19.	No. of transcripts in final assembly	22,402
20.	Size of final assembly (bp)	19,822,291
21.	N50 (bp)	1007
22.	Average transcript length (bp)	884.8
23.	Longest transcript length (bp)	5975
24.	Smallest transcript length (bp)	200
25.	GC%	45.69

## Data Availability

The data presented in this study are available in the article.

## Supplementary Materials

The following supporting information can be downloaded at: https://www.mdpi.com/article/10.3390/ijms24119212/s1.
