# Supplementary figures and images for: De Novo Transcriptome Profiling for the Generation and Validation of Microsatellite Markers, Transcription Factors, and Database Development for Andrographis paniculata
Source: Int J Mol Sci. 2023 May 24;24(11):9212. doi: 10.3390/ijms24119212 (PMC10252285; doi:10.3390/ijms24119212)

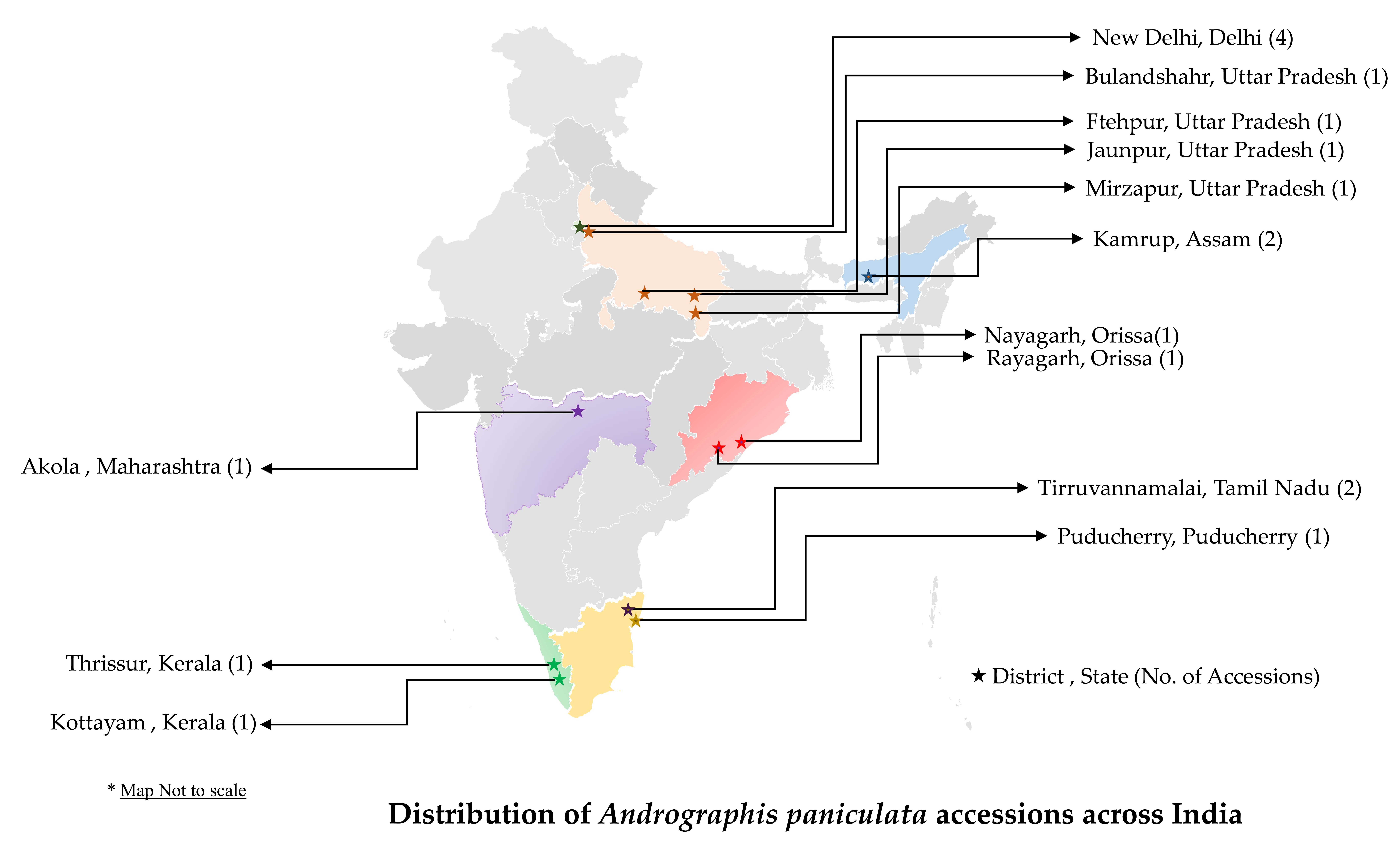

Supplement: Supplementary file 1 [file ijms-24-09212-s001.zip › Figure S6. Distribution of A. paniculata accessions across India.jpg]
